# Exploration of Risk Factors for Pancreatic Cancer and Development of a Clinical High-Risk Group Rating Scale

**DOI:** 10.3390/jcm12010358

**Published:** 2023-01-02

**Authors:** Qian Zhao, Yan Wang, Tianyu Huo, Furong Li, Lu Zhou, Yongliang Feng, Zhigang Wei

**Affiliations:** 1 Department of Epidemiology, School of Public Health, Shanxi Medical University, Taiyuan 030001, China; 2 Hepatobiliary and Pancreatic Surgery and Liver Transplantation Center, First Hospital of Shanxi Medical University, Taiyuan 030001, China; 3 Department of Pathology & Pathophysiology, School of Basic Medicine Shanxi Medical University, Taiyuan 030001, China

**Keywords:** pancreatic cancer, risk factors, high-risk groups, rating scale

## Abstract

(1) Background: There are few studies on people at high risk for clinical pancreatic cancer (PC). We aimed to explore the risk factors of PC and establish a scale for identifying high-risk populations of clinical PC. (2) Methods: We conducted a matched case-control study, retrospectively collecting demographic data and common clinical indicators from all subjects. Logistic regression was used to explore the risk factors of PC. Based on these factors, we created a high-risk population rating scale, which showed a higher diagnostic value. (3) Results: 385 cases and 428 controls were finally enrolled in our study. Multivariate analysis showed that body mass index (BMI) below 18.5 kg/m^2^ (OR 5.944, 95%CI: 1.759~20.084), smoking (OR 2.745, 95%CI: 1.555~4.844), new-onset diabetes (OR 5.239, 95%CI: 2.091~13.125), low high-density lipoprotein cholesterol (HDL-C) levels (OR 1.790, 95%CI: 1.044~3.069), and carbohydrate antigen 19-9 (CA19-9) levels no less than 35 U/mL (OR 160.328, 95%CI: 83.392~308.243) were associated with an increased risk of PC, whereas high total cholesterol (TC) levels were related to a lower risk of PC (OR 0.392, 95%CI: 0.211~0.730). The high-risk population scale, whose area under the receiver operating curve reached 0.948 (*p* < 0.001), showed a greater clinical diagnostic value. (4) Conclusions: Smoking history, new-onset diabetes, BMI, TC, HDL-C, and CA19-9 levels were associated with the risk of PC. The high-risk population rating scale might be used for early clinical PC screening.

## 1. Introduction

Pancreatic cancer (PC) is a malignant tumor of the digestive system with a five-year survival rate of only 10% [1]. According to global cancer data for 2020, PC incidence ranked 12th in malignancy incidence, while mortality ranked seventh in malignancy-related deaths [2]. According to the most recent American Cancer Society data, PC was expected to be the second leading cause of cancer death in the United States by 2030, with 60,403 new PC patients and 48,220 deaths in 2021 [3]. In China, the incidence rate of PC ranked tenth among malignant tumors, and the mortality rate ranked sixth among malignant tumor-related deaths, both of which were expected to rise in the coming years due to an aging population and lifestyle changes [4,5]. Currently, surgery is the sole method for “curing” PC. Nonetheless, PC is insidious, with atypical early symptoms, and the majority of patients are discovered at a late stage of the disease and cannot be treated surgically [6]. Studies have shown that the 5-year survival rate for patients with metastasis-free early-stage pancreatic cancer was 29%, compared to only 2.6% for those with distant metastases [7]. Consequently, identifying individuals at risk for various phases of PC is essential for the early diagnosis and treatment of PC.

Current epidemiological researches on PC risk factors focus mainly on lifestyle, previous diseases, and family history, while few studies have examined the factors that influence PC in conjunction with clinical indications [8,9]. Nevertheless, in clinical practice, we often use biochemical indicators to judge the disease comprehensively. Specific clinical indicators are essential for early detection, timely treatment, and prognosis improvement of the condition. In recent years, a large number of studies have shown that abnormal lipid levels are closely related to the malignant biological behavior of tumors. On the one hand, abnormal lipid metabolism provides energy for the rapid proliferation of tumor cells, and on the other hand, when cancer occurs, the physiological balance of lipid levels is disrupted, which also leads to disorders of lipid metabolism [10,11,12]. For example, related studies have pointed out that changes in lipid concentrations might be associated with the occurrence, development, and malignancy of colorectal cancer [13,14]. In a follow-up study of Korean adults, Kitahara et al. [15] found that TC levels were positively associated with the occurrence of rectal and prostate cancers in men and breast cancer in women. Ye et al. [10] showed that four lipid metabolism-related genes were significantly associated with the prognosis of pancreatic cancer patients, and they also pointed out that focusing on biological processes such as lipid metabolism that have an important impact on cancer development may introduce potential biomarkers for pancreatic cancer screening. CA19-9 was the most commonly used and current gold standard biological marker for PC, and in PC, CA19-9 was used as a biomarker, predictor, promoter, etc., as in [16,17]. Therefore, exploring the factors associated with the risk of PC in conjunction with clinical indicators could help determine the screening criteria for clinical PC.

In this research, we used a matched case-control study to retrospectively analyze the clinical data of 385 PC patients and 428 control individuals at the First Hospital of Shanxi Medical University from January 2016 to December 2021 in order to investigate the factors associated with the risk of PC and use them to develop a rating scale of PC high-risk population, with the goal of early detection and timely treatment of PC patients in clinical work.

## 2. Materials and Methods

### 2.1. Study Population

Patients at the First Hospital of Shanxi Medical University who had pathological or imaging examinations or clinical manifestations suggestive of PC from January 2016 to December 2021 were included in this study, but those who met any of the following criteria were excluded: (1) Patients with pathological types other than pancreatic ductal adenocarcinoma; (2) Patients with other malignant tumors in combination, and (3) Patients with incomplete data information. In addition, to serve as controls for the case group, sex- and age-matched individuals were randomly selected from the healthy population undergoing routine physical examinations at our hospital during the same period.

### 2.2. Study Design

**Figure 1:**
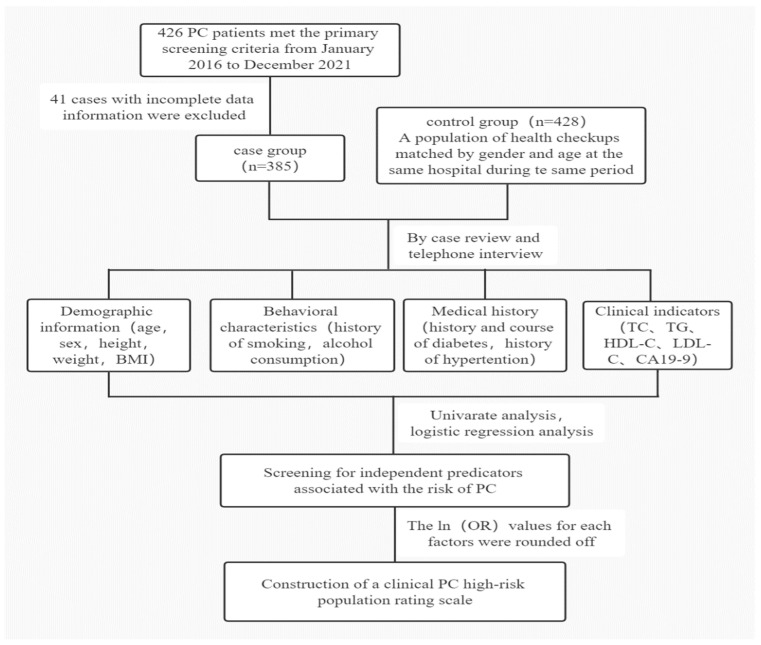
Study Design Flow Chart. PC: Pancreatic cancer; TC: Total cholesterol; TG: Triglycerides; HDL-C: High-density lipoprotein cholesterol; LDL-C: Low-density lipoprotein cholesterol; CA19-9: Carbohydrate antigen 19-9.

The following data were retrospectively collected by reviewing medical records and telephone follow-ups: age, gender, height, weight, body mass index(BMI), history of smoking, history of alcohol consumption, history of diabetes, history of hypertension, lipid indexes: total cholesterol (TC), triglycerides (TG), high-density lipoprotein cholesterol (HDL-C), low-density lipoprotein cholesterol (LDL-C) and tumor marker: carbohydrate antigen (CA19-9) levels. The factors associated with PC risk were first analyzed by univariate and multifactorial analyses. Then a high-risk population rating scale was constructed from the screened elements, and the clinical screening effect of the scale was validated and evaluated in the modeled population. The specific flow is shown in Figure 1.

### 2.3. Variable Definition

Lipid indicators referred to the level of biochemical indicators of our hospital: TC ≥ 5.20 mmol/L defined as high TC levels; TG ≥ 1.70 mmol/L defined as high TG levels; HDL-C ≤ 1.04 mmol/L defined as low HDL-C levels, and LDL-C ≥ 3.40 mmol/L defined as high LDL-C levels. In addition, according to the biochemical guidelines of our hospital, CA19-9 levels above or equivalent to 35 U/mL were considered abnormal. Moreover, there was no accepted definition of newly diagnosed and long-term diabetes. The majority of studies classified diabetes with a length of less than or equal to two years as new-onset diabetes and diabetes with a period of more than two years as long-term diabetes [18,19].

### 2.4. Data Analysis

SPSS22.0 and GraphPad Prism 8 were used for the statistical analysis of the data. If the measurement data followed a normal distribution, they were described by ($\bar{x}$ ± *s*) and compared between groups using the *t*-test; otherwise, they were described by *M* (*P*25~*P*75) and compared between groups using the Wilcoxon Rank-Sum test. The number of instances (percent) was employed to characterize count data, and groups were compared using chi-square or Fisher’s exact test. Multifactorial logistic regression analyses incorporated factors that were statistically different across groups in the univariate analysis to uncover independent predictors of pancreatic cancer risk.

By using logistic regression analysis, we obtained the odds ratio (OR) of each influencing factor. We then rounded the log odds (ln (OR)) of the screened predictors to the closest integer to build the scores for each component category to create the PC high-risk population rating scale. The total scores were calculated by adding the scores of each item. We used (ln (OR)) instead of odds ratios (OR) to calculate the scores for each element since the sum of (ln (OR)) was likely to be a better predictor of outcomes than the sum of (OR)s [20,21]. In addition, Medcalc20.010 was used to plot the receiver operating curve (ROC), and the area under the curve (AUC) was utilized to evaluate the diagnostic results’ validity: a c-statistic coefficient higher than 0.8 indicated a strong model. *p*-values (two-sided) less than 0.05 were considered statistically significant.

## 3. Results

### 3.1. Basic Information of the Study Subjects 

This study comprised 385 PC (case group) patients with a mean age of onset of (64.75 ± 10.65) years. In the case group, there were 260 males and 125 females, for a male-to-female sex ratio of 2.08:1. In addition, 428 controls (control group) were chosen from the healthy physical examination population throughout the same time, with a mean onset age of (64.28 ± 10.65) years. There were 299 males and 129 females in the control group for a male-to-female ratio of 2.32:1. Regarding age and gender, there were no statistical differences between the two groups (*p* = 0.534, *p* = 0.47).

**Figure 2:**
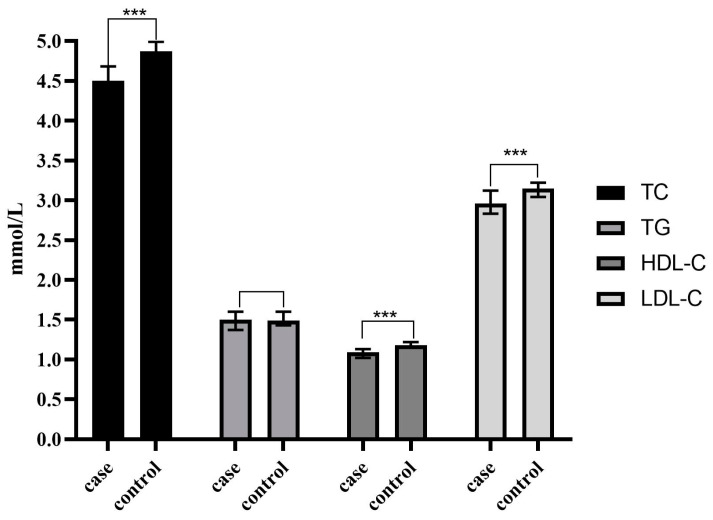
Serum lipid difference between case and control groups in our study. TC: Total cholesterol; TG: Triglycerides; HDL-C: High-density lipoprotein cholesterol; LDL-C: Low-density lipoprotein cholesterol; CA19-9: Carbohydrate antigen 19-9; *** *p* < 0.001.

In terms of diabetes history, the proportion of patients with combination diabetes in the case group was higher than that in the control group (27.5% vs. 15.0%, *p* < 0.001). The proportion of hypertension patients in the two groups did not differ statistically (*p* = 0.530). The case group’s mean BMI levels were lower than that of the control group (*p* < 0.001), whereas CA19-9 levels were greater (*p* < 0.001) (Table 1). Besides, as shown in Table 1 and Figure 2, the mean levels of TC, HDL-C, and LDL-C in the case group were lower than those in the control group (*p* < 0.001).

### 3.2. Univariate Analysis of Risk Factors for PC 

Univariate analysis of potential PC risk variables found that BMI (*p* < 0.001), smoking history (*p* < 0.001), the status of diabetes (*p* < 0.001), TC (*p* < 0.001), HDL-C (*p* < 0.001), LDL-C (*p* < 0.001), and CA19-9 (*p* < 0.001) levels were associated with PC prevalence (Table 2).

### 3.3. Multi-Factor Analysis of Risk Factors for PC

To screen out independent predictors linked with the likelihood of PC prevalence, we performed a supplementary logistic regression analysis on the above statistically significant single components. Multifactorial analysis revealed that BMI levels below18.5 kg/m^2^ (OR 5.994, 95%CI:1.759~20.084), history of smoking (OR 2.745, 95%CI:1.555~4.844), low HDL-C levels (OR 1.790, 95%CI:1.044~3.069), new-onset diabetes(OR 5.239, 95%CI:2.091~13.125), and CA19-9 ≥ 35 U/mL (OR 160.328, 95%CI:83.392~308.243) were associated with an increased risk of PC, whereas high TC levels(OR 0.392, 95%CI:0.211~0.730) were associated with a decreased risk of PC (Table 3).

### 3.4. Construction of a Clinical PC Screening Score Scale for High-Risk Groups

To visualize the results of the study and make them more readily applicable to clinical practice, we developed a clinical PC screening scale comprised of six factors: BMI, smoking history, TC, HDL-C, diabetes status, and CA19-9 (Table 3). The scores for each factor were rated after rounding its (lnOR)) to the nearest absolute number. Table 4 shows the individual scores for each factor, with a total project score of 12.

### 3.5. Validation and Evaluation of the Screening Scoring Criteria for Clinical PC Risk Populations

**Figure 3:**
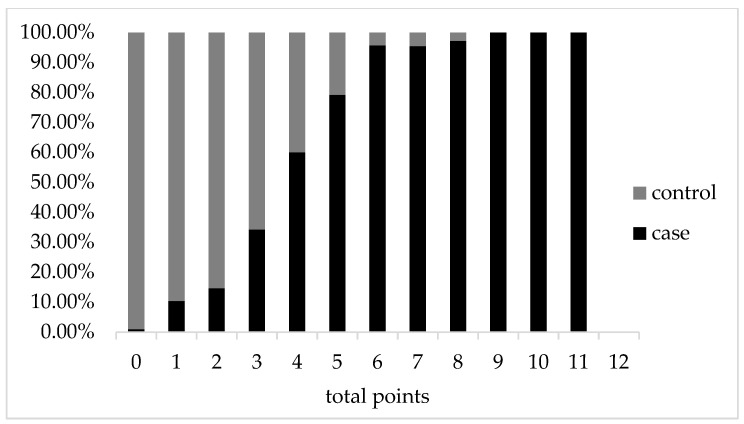
Composition of each hazard score in the modeled population. ■ represents the composition ratio of patients in the case group at this score, and 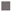 represents the composition ratio of the control group at the same score. The total score was 12 points.

**Figure 4:**
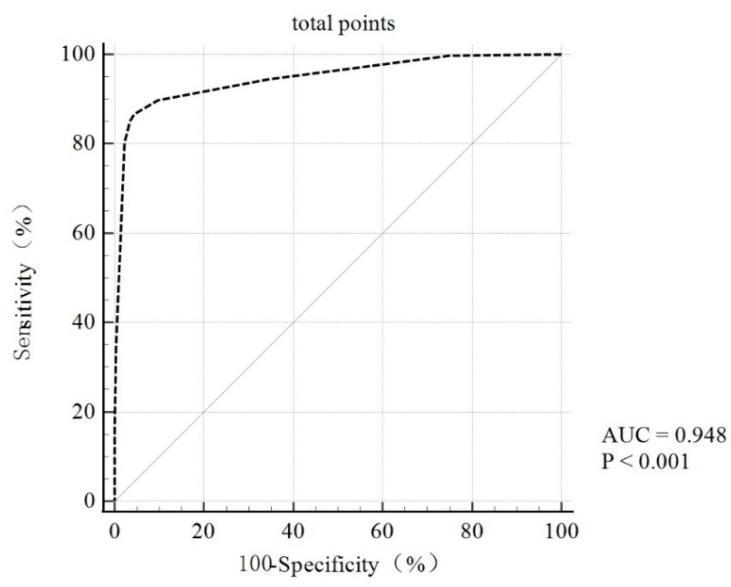
ROC curve of the PC screening scale. ROC: The receiver operating curve; AUC: The area under the curve.

The case group was related to higher risk scores, as shown in Figure 3, and the proportion of patients in the case group with the same score tended to increase as the overall score increased (*p* < 0.001). The Omnibus test of model coefficients revealed that the model was statistically significant (*p* < 0.001) overall, and the Hosmer–Lemeshow goodness-of-fit results revealed that the rating scale was a good fit (*p* = 0.183). Furthermore, for this rating scale, we plotted the ROC curve, which had an area under the curve of 0.948 (*p* < 0.001), indicating that it had a good predictive value. A total score greater than 3 was the ideal cut-off value for this scale for evaluating patients at high risk for clinical PC (Figure 4).

## 4. Discussion

PC has a high fatality rate, and early detection is challenging [9,22]. Currently, there is no comprehensive PC screening program in China. In conjunction with clinical indicators, relatively few PC risk factors have been investigated in previous studies. On the basis of general influencing variables, our study explored PC risk factors in conjunction with clinical indicators and produced a relatively comprehensive clinical PC risk group scale, which was useful for the early diagnosis of PC patients in clinical work. Our study suggested that BMI levels below 18.5 kg/m^2^, history of smoking, new-onset diabetes, low HDL-C levels, and CA19-9 no less than 35 U/mL were related to an increased risk of PC, while high TC levels were associated with a decreased risk of PC. Based on these results, the clinical PC high-risk population rating scale was well fitted in the modeled population, and the scale had solid predictive value when used for screening of the clinical PC high-risk population.

There is currently no consensus regarding the relationship between BMI and PC [23]. A study by Jacobs et al. [24] found that higher BMI levels were associated with an increased risk of PC. In addition, in a 15-year follow-up study, Arjani et al. [25] found that high BMI and a BMI trajectory leading to overweight or obesity in adulthood were positively associated with PC, with stronger associations in early-onset obesity and in the male population. Avoiding being overweight throughout the adult life course may prevent PC. In contrast, a prospective study of Chinese Singaporeans found that among former smokers, the risk of PC was 1.99 times higher among those with BMI levels of below 18.5 kg/m^2^ than among those with BMI levels of 21.5~24.4 kg/m^2^ (HR 1.99, 95% CI:1.03~3.84), whereas there was no association between BMI and PC risk among never smokers [26]. In our study, the case group had lower BMI levels than the control group. At the same time, the results of the multifactorial analysis showed that BMI levels below 18.5 kg/m^2^ were associated with an increased risk of PC. This finding may be attributable to the fact that this was a case-control study, and the majority of patients had advanced PC at the time of clinical diagnosis. Patients with advanced PC frequently experienced considerable weight loss due to cachexia.

Smoking is the only factor confirmed to have a causal link with PC by the International Agency for Research on Cancer [27]. Our study also found smoking to be an independent risk factor for PC. A genome-wide association investigation of European ancestry individuals identified a susceptibility gene at 2q21.3 that significantly affected the risk of PC by smoking status and revealed that never-smokers had the lowest risk of PC compared to those with a smoking history [28].

Recently, the relationship between diabetes and PC has been a prominent focus of study [29,30]. A meta-analysis of 26 case-control studies revealed that the risk of PC was inversely proportional to the duration of diabetes, with newly diagnosed diabetic individuals having the highest risk of PC [31]. New-onset diabetes was related to an elevated risk of PC in our analysis, whereas the association between long-term diabetes and PC was not statistically significant, suggesting that new-onset diabetes may represent an early clinical manifestation of PC.

It is believed that lipid metabolism abnormalities are related to several malignancies, including PC [10,32]. However, there is no conclusive evidence of the changes in lipid levels in PC patients. A cohort study by Kuzmickiene et al. [33] revealed no statistical link between TC levels and PC risk; however, a two-center retrospective investigation from China reported greater TC levels in PC patients than in non-PC patients and concluded that a high lipid diet increased the development of PC [34]. Consistent with the findings of Strohmaier et al. [35] and Bo et al. [36], our analysis revealed that elevated TC levels were related to a decreased risk of PC. On the one hand, preclinical cancer may block TC’s metabolism. On the other hand, it was hypothesized that the inverse relationship between cancer and TC could be explained by a competitive mechanism, in which some patients with high levels of TC died prior to developing cancer for reasons such as cardiovascular disease, thereby reducing the proportion of patients with high TC levels in PC [35,37]. HDL-C has antioxidant properties and aids in cholesterol clearance. In comparison to LDL-C levels, high HDL-C levels usually indicate good health. Patel et al. [38] found that lower HDL-C levels were associated with an increased cancer risk, which was consistent with our findings and could be explained by the fact that cancer status affected serum HDL-C levels.

In our study, the positive rate of CA19-9 in the case group was 84.4%, and the level of CA19-9 no less than 35 U/mL was positively correlated with the risk of PC, which to some extent indicated the importance of using CA19-9 as a diagnostic indicator for PC. CA19-9 is the only recognized serologic diagnostic marker for PC at the moment. Nonetheless, the diagnostic specificity of CA19-9 is compromised by inflammation, false positives in non-PC states, and false negatives in Lewis antigen-negative individuals [16,39,40]. The discovery of new serological markers, when combined with CA19-9 and other tumor markers to perform the test, may aid in the early detection of PC.

Exploring the factors linked with the risk of PC utilizing general factors and common clinical indicators can aid in developing clinical screening criteria for PC, which can aid physicians in the early detection of PC patients [41]. On the basis of these contributing factors, we developed a clinical PC high-risk population rating scale that had an excellent predictive value in the modeled population. Currently, imaging remains the most critical screening method for PC, and imaging is frequently expensive [9,22]. Most families are financially burdened by annual or semiannual imaging. Suppose we can identify the group of patients at high risk for PC that requires this test before the imaging test. In this situation, we can lessen the financial burden on the family while potentially detecting more patients with clinical PC at an early stage. The absence of imaging information in our scale and the presence of more readily identified and accessible characteristics linked with the likelihood of developing PC could be used clinically to screen individuals at high risk of clinical PC before imaging.

## 5. Conclusions

In conclusion, we constructed a clinical PC high-risk population score scale using some general factors and routine clinical indicators that were easier to identify and obtain. It had some clinical use because of its lower screening cost. At the same time, the scale also had some drawbacks. For example, because of the case-control study used, some factors could only be shown to correlate with PC, and the exploration of the causal relationship was to be verified in prospective studies.

## Figures and Tables

**Table 1:** Baseline characteristics of the study population.

Variables	Case (*n* = 385)	Control (*n* = 428)	*t/Z/χ* ^2^	*p* Value
Age, y	64.75 ± 10.65	64.28 ± 10.65	*T* = 0.622	0.534
Sex [*n*, (%)]			*χ*^2^ = 0.511	0.475
Male	260(67.5)	299(69.9)		
Female	125(32.5)	129(30.1)		
Smoking history [*n*, (%)]			*χ*^2^ = 29.559	<0.001 ***
Yes	137(35.6)	80(18.7)		
No	248(64.4)	348(81.3)		
Drinking history [*n*, (%)]			*χ*^2^ = 2.645	0.104
Yes	82(21.3)	72(16.8)		
No	303(78.7)	356(83.2)		
Status of diabetes [*n*, (%)]			*χ*^2^ = 38.026	<0.001 ***
No diabetes	279(72.5)	364(85.0)		
New-onset diabetes	62(16.1)	15(3.5)		
Long-term diabetes	44(11.4)	49(11.4)		
History of hypertension [*n*, (%)]			*χ*^2^ = 0.394	0.530
Yes	126(32.7)	149(34.8)		
No	259(67.3)	279(65.2)		
BMI (kg/m^2^)	21.75 ± 3.24	25.36 ± 3.23	*T* = −15.912	<0.001 ***
TC (mmol/L)	4.50(3.56~4.75)	4.87(4.15~5.58)	*Z* = −7.870	<0.001 ***
TG (mmol/L)	1.50(1.04~1.71)	1.49(1.09~2.04)	*Z* = −1.806	0.071
HDL-C(mmol/L)	1.09(0.82~1.14)	1.18(1.04~1.39)	*Z* = −9.260	<0.001 ***
LDL-C(mmol/L)	2.96(2.24~3.16)	3.15(2.64~3.71)	*Z* = −5.975	<0.001 ***
CA19-9(U/mL)	235.17 (62.3~1026.10)	5.61 (3.50~9.00)	*Z* = −20.724	<0.001 ***

BMI: Body mass index; TC: Total cholesterol; TG: Triglycerides; HDL-C: High-density lipoprotein cholesterol; LDL-C: Low-density lipoprotein cholesterol; CA19-9: Carbohydrate antigen 19-9; *** *p* < 0.001.

**Table 2:** Univariate analysis of risk factors for PC.

Variables	Case (*n* = 385)	Control (*n* = 428)	*t/χ* ^2^	*p* Value
BMI [*n*, (%)]			*χ*^2^ = 52.151	<0.001 ***
<18.5	60(15.6)	7(1.6)		
≥18.5	325(84.4)	421(98.4)		
Smoking history [*n*, (%)]			*χ*^2^ = 29.559	<0.001 ***
Yes	137(35.6)	80(18.7)		
No	248(64.4)	348(81.3)		
Drinking history [*n*, (%)]			*χ*^2^ = 2.645	0.104
Yes	82(21.3)	72(16.8)		
No	303(78.7)	356(83.2)		
Status of diabetes [*n*, (%)]			*χ*^2^ = 38.026	<0.001 ***
No diabetes	279(72.5)	364(85.0)		
New-onset diabetes	62(16.1)	15(3.5)		
Long-term diabetes	44(11.4)	49(11.4)		
History of hypertension [*n*, (%)]			*χ*^2^ = 0.394	0.530
Yes	126(32.7)	149(34.8)		
No	259(67.3)	279(65.2)		
TC [*n*, (%)]			*χ*^2^ = 52.069	<0.001 ***
<5.2 mmol/L	324(84.2)	263(61.4)		
≥5.2 mmol/L	61(15.8)	165(38.6)		
TG [*n*, (%)]			*χ*^2^ = 0.030	0.864
<1.70 mmol/L	228(59.2)	256(59.8)		
≥1.70 mmol/L	157(40.8)	172(40.2)		
HDL-C [*n*, (%)]			*χ*^2^ = 37.378	<0.001 ***
>1.04 mmol/L	208(54.0)	319(74.5)		
≤1.04 mmol/L	177(46.0)	109(25.5)		
LDL-C [*n*, (%)]			*χ*^2^ = 38.752	<0.001 ***
<3.40 mmol/L	316(82.1)	267(62.4)		
≥3.40 mmol/L	69(17.9)	161(37.6)		
CA19-9 [*n*, (%)]			*χ*^2^ = 552.587	<0.001 ***
<35 U/ml	60(15.6)	415(97.0)		
≥35 U/ml	325(84.4)	13(3.0)		

BMI: Body mass index; TC: Total cholesterol; TG: Triglycerides; HDL-C: High-density lipoprotein cholesterol; LDL-C: Low-density lipoprotein cholesterol; CA19-9: Carbohydrate antigen 19-9; *** *p* < 0.001.

**Table 3:** Logistic regression analysis of risk factors for PC.

Variables	*β*	*S. E*	*Wald χ^2^*	*p* Values	OR (95%CI)
BMI < 18.5 kg/m^2^					
No					1
Yes	1.782	0.621	8.232	0.004 **	5.944(1.759~20.084)
Smoking history					
No					1
Yes	1.010	0.290	12.141	<0.001 ***	2.745(1.555~4.844)
High TC levels					
No					1
Yes	−0.936	0.317	8.737	0.003 **	0.392(0.211~0.730)
Low HDL-C levels					1
No					1
Yes	0.582	0.275	4.476	0.034 *	1.790(1.044~3.069)
Status of diabetes			12.500	0.002 **	
No diabetes ^↑^					1
New-onset diabetes	1.656	0.469	12.493	<0.001 ***	5.239(2.091~13.125)
Long-term diabetes	0.204	0.406	0.252	0.615	1.226(0.554~2.716)
CA19-9 ≥ 35 U/ml					
No					1
Yes	5.077	0.334	231.756	<0.001 ***	160.328(83.392~308.243)
Constant term	−2.401	0.235	104.013	<0.001	

BMI: Body mass index; TC: Total cholesterol; TG: Triglycerides; HDL-C: High-density lipoprotein cholesterol; LDL-C: Low-density lipoprotein cholesterol; CA19-9: Carbohydrate antigen 19-9; CI: Confidence interval; ^↑^ Reference group for comparisons; * *p* < 0.05; ** *p* < 0.01; *** *p* < 0.001.

**Table 4:** Determination of the scores for each factor in the PC screening scale.

Variables	OR (95%CI)	*p* Values	ln (OR)	Scores
BMI < 18.5 kg/m^2^		0.004 **		
Yes	5.944(1.759~20.084)		1.782	2
No	1			0
Smoking history		<0.001 ***		
Yes	2.745(1.555~4.844)		1.013	1
No	1			0
High TC levels		0.003 **		
Yes	0.392(0.211~0.730)		−0.936	0
No				1
Low HDL-C levels		0.034 *		
Yes	1.790(1.044~3.069)		0.582	1
No				0
CA19-9 ≥ 35 U/mL		<0.001 ***		
Yes	160.328(83.392~308.243)		5.077	5
No				0
Status of diabetes		0.002 **		
No diabetes				0
New-onset diabetes	5.239(2.091~13.125)	<0.001 ***	1.656	2
Long-term diabetes	1.226(0.554~2.716)	0.615		0

BMI: Body mass index; TC: Total cholesterol; HDL-C: High-density lipoprotein cholesterol; CA19-9: Carbohydrate antigen 19-9; CI: Confidence interval; * *p* < 0.05; ** *p* < 0.01; *** *p* < 0.001.

## Data Availability

The datasets generated during and/or analyzed during the current study are available from the corresponding author upon reasonable request.
